# Computational characterization and control of electrical conductivity of nanowire composite network under mechanical deformation

**DOI:** 10.1038/s41598-018-34992-6

**Published:** 2018-11-09

**Authors:** Jinyoung Hwang, Hiesang Sohn, Sang Hyun Lee

**Affiliations:** 1 School of Electronics and Information Engineering, Korea Aerospace University, Goyang-si, 10540 Korea; 2 Department of Chemical Engineering, Kwangwoon University, Seoul, 01897 Korea; 3 School of Electrical Engineering, Korea University, Seoul, 02841 Korea

## Abstract

Quantitative models to predict the electrical performance of 1-D nanowire (NW) composite networks under external deformation such as bending and patterning are developed by Monte-Carlo based computations, and appropriate solutions are addressed to enhance the tolerance of the sheet resistance (R_s_) of the NW networks under the deformation. In addition, several strategies are employed to improve further the robustness of the sheet resistance against the network deformation. In the case of bending, outstanding bending durability of a hybrid NW network coated on a 2-D sheet is confirmed with a numerical model, and a network of NWs aligned unidirectionally toward bend axis is introduced to alleviate the sheet resistance degradation. In the case of a narrowly patterned channel, the conductivity enhancement of a network of NWs aligned in parallel to the channel with reduced channel is validated, and a network made with two types of NWs with different lengths is suggested to enhance the tolerance of the electrical conductivity. The results offer useful design guidelines to the use of the 1-D NW percolation network for flexible transparent conducting electrodes.

## Introduction

Flexible transparent conducting electrodes (TCEs) are essential components for various modern electronic devices including wearable sensors, flexible displays, batteries and solar cells, etc^1–3^. Indium-tin-oxide (ITO), although most popularly used for TCEs owing to its high electrical conductivity and optical transmittance, is not suitable for flexible TCEs because of its limited flexibility^4^. Alternatives for flexible TCEs, which have high optical transparency, electrical conductivity, and flexible mechanical property, have recently been introduced to replace brittle ITO. For example, percolation networks of one-dimensional (1-D) nanostructures (nanowires (NWs), nanorods, nanotubes, etc.)^5–17^, 2-D materials (graphene and MnO_2_, etc.)^18–20^ and conducting polymers (PEDOT:PSS, polyaniline, etc.)^21–23^ are utilized in a wide range of electronic devices^5–15,18–23^. In particular, percolation networks of 1-D nanostructures have been extensively investigated for applications of flexible TCEs^5–15^. A random network of NWs shows remarkable electrical and optical properties: the sheet resistance (R_s_) and transmittance are beyond about 10 Ω/sq and 90%, respectively, thereby providing much improved performance than ITO^4^. Such outstanding performance can be achieved with ultra-high aspect ratio NWs of highly conductive NW materials such as carbon nanotube (CNT), gold, silver (Ag), copper, and their alloys^5–11^. Furthermore, high-quality NWs can be synthesized via low-cost and scalable wet-based process, and a random NW network can be fabricated by coating a dispersed NW solution on a flexible substrate using slot-die method, bar-coating, and spin coating, and spray coating process^1,3,7,24,25^. In addition, a number of studies have addressed the improvement of the electrical and optical performance of the NW network. The sheet resistance of the NW network enhances as the areal density of the NW increases. At a given NW areal coverage, the performance can be improved by increasing the aspect ratio of the NW^26^. If the network is constructed using NWs of various lengths together, a small number of high aspect ratio NWs (10% of total areal coverage of NWs) yield a drastic reduction of R_s_^27^. Furthermore, the reduced contact resistance between NWs and the decrease in the resistivity of the NW enhance the sheet resistance of networks without sacrificing the optical performance^1^.

The flexible TCEs made up of the NW percolation network are primarily used in electrode patterns for touch screen panels, organic light emitting diodes and solar cells^1–3,5–10^. In such applications, the NW network is subject to severe mechanical distortion during the operation of devices and is, in general, patterned into submicroscale channels, thereby incurring noticeable debilitation in the electrical conductivity of TCEs^28^. The sheet resistance of the NW network distorted by external deformation such as bending or patterning increases significantly as compared to the resistance of the original network. In the NW network coated on a polymer substrate under bending deformation, the NWs have relatively weak yield strength and are easy to break down, while the substrate can bend. Thus, a percolating path containing such NWs becomes disconnected and, in consequence, the network resistance increases. Furthermore, in the NW network patterned with the feature size relatively smaller than the NW length, the portion of a NW placed off the channel region is cut by patterning, and a large number of the remaining NWs in the channel are shortened. Thus, the effective average length of the NW diminishes while the areal coverage remain unchanged, thereby increasing the sheet resistance of the network. This resistance increase by deformation poses a unique challenge in 1-D random networks and has not appeared among other TCEs with 2-D and bulk materials. Experimental attempts have been made to overcome fundamental limitations and have shown enhanced performance under severe conditions. To be specific, a NW network encapsulated with 2-D materials presents excellent mechanical durability at various configuration of bending test^29^. Also, a NW network directly assembled along a narrow channel direction shows enhanced conductivity with a reduced width of the channel^30^.

Numerical studies on the electrical conductivity of CNT/polymer and Ag NWs/elastomer nanocomposite under the external strain have been reported^31–34^. Those works demonstrate that the relocation of wires in a 3D matrix causes the change of tunneling effect between two wires, thereby leading to the modification of the contact resistance between those wires. Subsequently, this change gives rise to the variation of sheet resistance of the nanocomposite.

Unlike a 3D composite network where the distance between NWs is prone to the disruption under tensile strain and a significant change in the tunneling resistance among NWs occurs, the modification of the tunneling effect is not a critical factor in the case of a 2D network. Instead, Lihua Jin *et al*. demonstrated resistance characteristics of a CNT network under tensile strain, showing that reorienting and sliding CNTs under tensile strain increases the resistance of the network and that buckling CNTs during strain release process leads to a hysteresis^34^. Yet, these methods are not straightforwardly extended to account for the resistance change of the NW networks used for TCEs, where the component NWs are made with stiff materials (e.g., semiconductor, oxide, and metal nanowires) and their yield strengths are much less than CNTs. In such cases, the NWs coated on a flexible substrate tend to break down rather than becoming elongated or bent under a range of tensile strain applied in operation of the devices. Most of currently available TCEs are made based on such NW networks, and the understanding of the impact on the electrical properties by the wire breakage under strain incorporated in the 2-D model is necessary.

This work develops quantitative models of the electrical conductivity of a 1-D NW composite network under external deformation via Monte-Carlo based computation. Based on computational models, the tolerance of the resistance under the external deformation such as bending and patterning is investigated with respect to various factors, and appropriate solutions are developed for the performance improvement. In addition, experimental attempts previously developed to overcome fundamental limitations of the NW network is studied using the model, and the improved conductivity under external deformation is confirmed. Finally, new methods are also suggested to alleviate the sheet resistance degradation of the NW network under the external deformation, and the performance enhancement is demonstrated in a computational way.

## Results and Discussion

The Monte-Carlo simulation is described briefly. The details are contained in Supplementary Information S1. To predict the sheet resistance (R_s_) of a 1-D nanowire (NW) percolation network, a random instance of the NW network is generated in a square domain by placing rectangular rods representing NWs such that the width and length of a rod correspond to the diameter and length of a cylindrical NW, respectively. Two-dimensional center coordinates and angle with respect to horizontal axis are chosen at random to characterize a randomly placed rectangular rod. Upon completion of the placement, the connection status of the network is examined by checking connected rods in the network form a conducting path from one end to the other end of the square domain. The test of the connectivity between two rods consists in calculating the shortest distance between the rods. If the shortest distance between two rods is less than the width of the rod, the rods are considered to touch with each other. The connectivity information is gathered by clustering analysis for exploring percolating clusters that traverse across the square domain. A percolating cluster that expands gradually from the left end to the right end becomes a conducting path. The overall resistance of the network is obtained based on Kirchhoff’s current law (KCL) applied to the network with a 1 V voltage source by considering two types of the resistance arising from inherent NW resistance (R_NW_) and contact resistance between two NWs (R_C_). The KCL formulates a system of linear equations with respect to the voltage drop at every contact in the conducting path, and the total current flow flowing across the square domain is calculated from the solution. From the assumption of the 1 V voltage source, the R_s_ of the network is estimated from the reciprocal of the obtained total current.

The parameters of a NW considered in the simulation are set according to experimentally measured properties of a currently available Ag NW. A NW is geometrically modeled as a cylinder with the diameter of 30 nm, the length ranging from 10 to 40 μm, and the resistivity of 3.18 × 10^-8^ Ω·m^35,36^. The contact resistance, denoted by R_c_, for two NWs in contact is set to 500 Ω, which is slightly smaller than the previously used value of the contact resistance between two Ag NWs to reflect the improvement in fabrication technology^27^. The value of R_c_ is set to be constant in the NW network since the tunneling resistance in the 2-D NW network does not undergo a large variation under the external deformation^27,34^.

**Figure 1:**
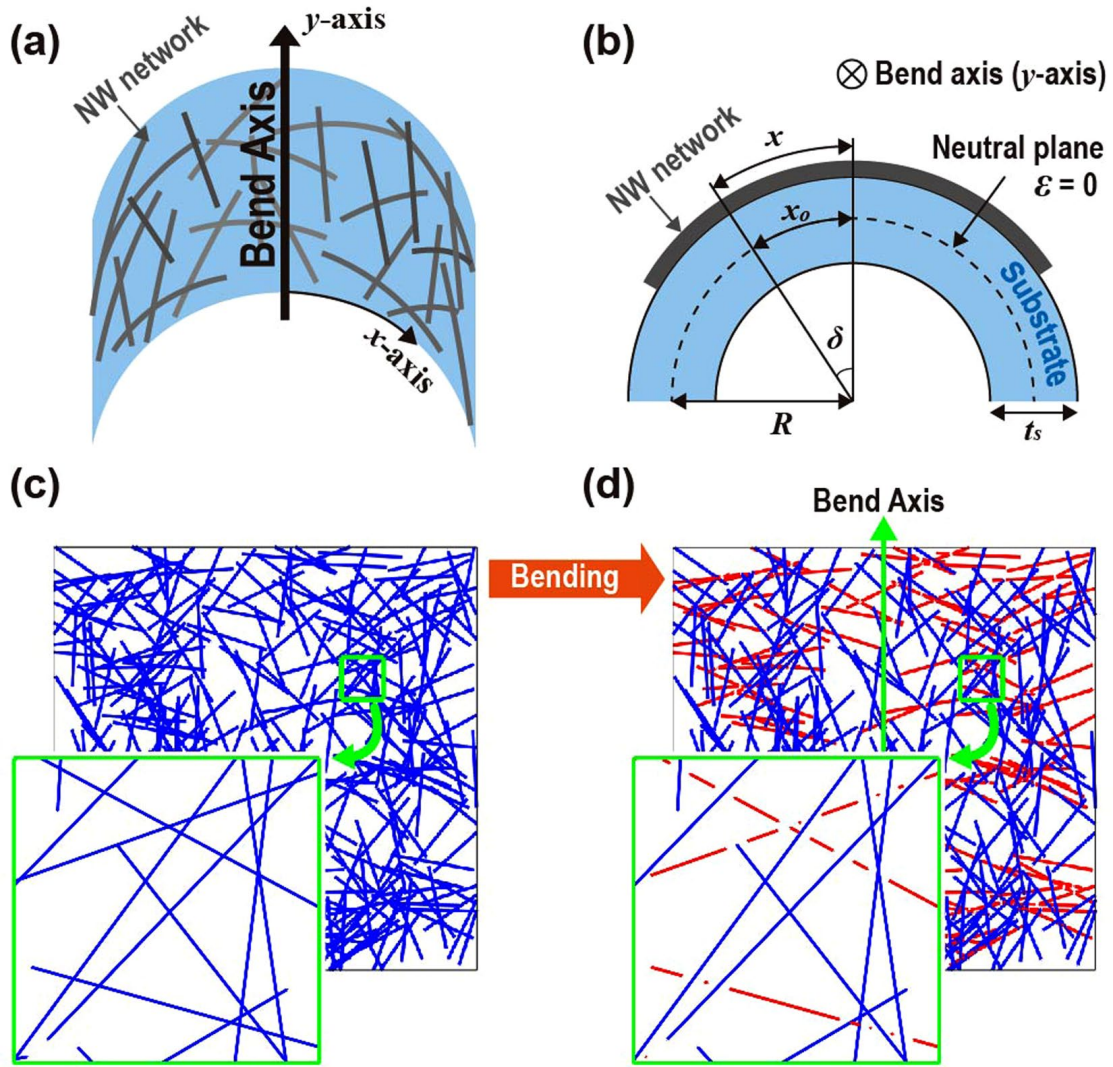
(**a**) A schematic description of a NW network under outward bending. In this model, the network coated on a flexible substrate is bent along the bend axis (*y*-axis). (**b**) A cross-sectional view of a NW network on an outwardly bent flexible substrate is shown. The neutral plane of the substrate is positioned at half of the film thickness (represented in a dashed curve), and the thickness of the substrate (*t*_*s*_) is much larger than that of the NW network. (**c**) An instance of NW network generated in the Monte-Carlo simulation. (**d**) The simulation image represents the NW network in (**c**) that is subjected to outward bending. The broken NWs due to bending deformation greater than the yield strain are indicated in red lines.

Figure 1(a) illustrates the model for the Monte-Carlo simulation to examine the effect of bending on the sheet resistance of the NW network. As presented in Fig. 1(b), the NW network is coated on a homogeneous flexible substrate with a thickness (*t*_*s*_) of 50 μm. The resulting NW network is much thinner than the substrate. The bending strain induced in the top-coated layer can be approximated by ${\rm{\varepsilon }}\approx {t}_{s}/2R$, where *R* represents the radius of curvature of the bending. Figure S2 depicts the top view of Fig. 1(b) and shows that the substrate bending leads to the extension of the NW in *x*-direction only. As a result, on the condition of a fixed *R*, the extended length of the NW depends on the angle (*θ*_0_) between *x*-axis (normal to the bend axis) and the NW, resulting in varying strain induced in the NW depending on *θ*_0_. The strain on the NW being ${t}_{s}/2R$ when *θ*_0_* = *0 decreases as *θ*_0_ increases and will eventually become zero when *θ*_0_ approaches to 90°. It can be expressed by the following equation. (The detailed derivation of the equation (1) is presented in Supplementary Information S2)

$${\rm{\varepsilon }}({\theta }_{0})=\sqrt{{(\frac{2R+{t}_{s}}{2R})}^{2}{\cos }^{2}{\theta }_{0}+{\sin }^{2}{\theta }_{0}}-1$$

Along with the length extension, the reduction in the diameter of the NW is also considered due to the strain-induced length extension assuming the conservation of the NW density, thus resulting in increased resistance of the NW. In addition, the yield strain of the NW is set to 1.5% in accordance with experimental results for Ag NWs with a diameter of 30 nm^37^. After obtaining a single random instance of the NW network seen in Fig. 1(c), the NWs under strain above the yield strain (*ε* = 1.5%) fracture at every contact where the NW crosses other NWs as depicted in the inset of Fig. 1(d). According to Fig. 1(d), the NWs with smaller *θ*_0_ are broken (represented in red lines) due to a large strain, while the NWs with larger *θ*_0_ remain intact (represented in blue lines).

**Figure 2:**
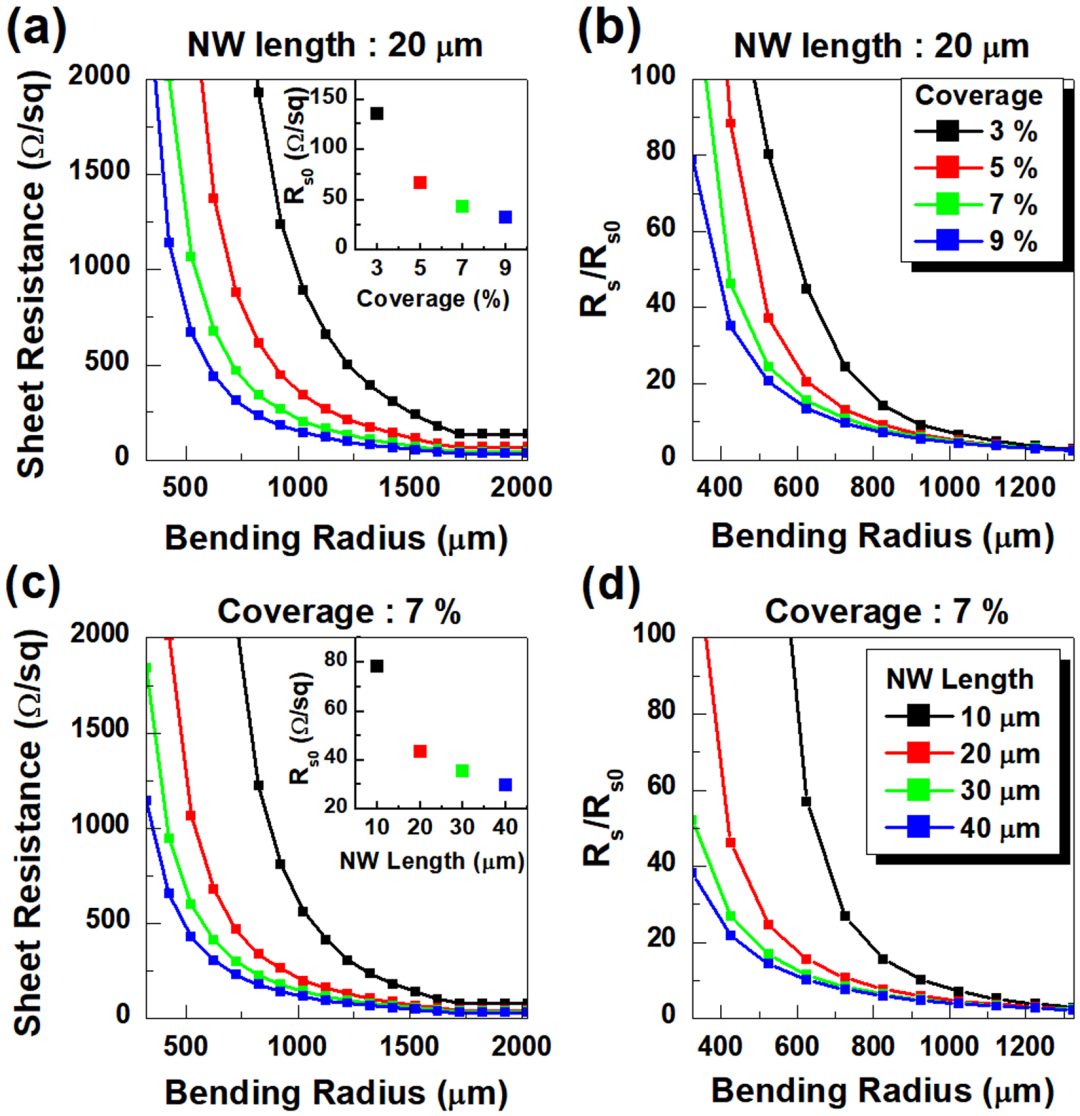
(**a**) Sheet resistance (R_s_) of the networks with 20 μm NWs as a function of bending radius (*R*) with respect to the NW areal coverage. (inset) Original R_s_ values (R_s0_) of the NW networks corresponding to the NW areal coverage obtained before applying the bending strain. Each data point in the inset represents the R_s0_ and the corresponding NW areal coverage of the network used in the simulation. (**b**) Relative change of R_s_ (R_s_/R_s0_) obtained from the data in (**a**). (**c**) R_s_ of the network with an NW areal coverage of 7**%** as a function of bending radius with respect to the NW length. (inset) R_s0_ and the corresponding NW length of the networks used in the simulation. (**d**) R_s_/Rs_0_ *versus* the bending radius obtained from the data in (**c**).

Figure 2 plots the changes in the sheet resistance of the NW network corresponding to the bending radius (*R*) with the variation in the areal coverage and the length of the NW. The change in the sheet resistance can be characterize by the ratio of the sheet resistance before (R_s0_) and after (R_s_) applying bending strain, i.e. R_s_/R_s0_. Note that the sheet resistance is calculated based on the electrical current flowing in the direction normal to the bend axis. The sheet resistance and its relative variation from the original value without bending strain (R_s_/R_s0_) increase sharply with decreasing *R* below a certain value under 2 mm, which agrees well with experimental studies^31,38–41^. Furthermore, R_s_ and R_s_/R_s0_ of the percolation network of the NWs with the same geometry (diameter = 30 nm and length = 20 μm), corresponding to the results in Fig. 2(a,b), respectively, improve the bending strain tolerance as the areal coverage of the NW network increases. In the case of 7% NW areal coverage shown in Fig. 2(c,d), a network made with longer NWs has higher robustness to the bending deformation. With a fixed areal coverage, a network consisting of longer NWs has a lower sheet resistance since it contains more number of percolating paths. Long NWs can form a percolating path with a small number of the wire, and the resulting percolating path has a small number of contacts, which lead to a low resistance. Thus, the total number of percolating paths also increase. Such a configuration can also be interpreted as a large number of low-resistance resistors connected in parallel. On the other hand, a large number of short NWs form a percolating path, and the resulting path has many contacts which cause the increased resistance per path. As a result, the network may contain a relatively small percolating cluster and can be thought of as a parallel connection of a few high-resistance resistors. If both networks are bent to the same extent, the same fraction of NWs break down in both networks since the bending strain induced on a NW depends on the angle between the longitudinal direction of the NW and the bend axis. The NWs with the angle out of the range corresponding to elastic deformation break down. Since, in both networks, the NWs are distributed uniformly at random, the fraction of NWs having the angle in the range is identical, thus undergoing the same fraction of the NW breakage. However, a long-NW network with small number of NWs forming a percolating path has smaller probability of losing a percolating path than a short-NW network, and the loss of small number of resistors in the parallel connection among many resistors causes a small increase of the total sheet resistance. Therefore, a long-NW network has high robustness against the bending strain.

To characterize the impact of the shape of the domain on the sheet resistance, various configurations of the bending axis angle with respect to a square-shaped domain are examined as presented in Supplementary Information S4. The sheet resistance change becomes less sensitive to bending strain as the bend axis rotates toward the direction parallel to the current flow in the NW network. This results from the fact that less tensile strain is exerted to the NW aligned further along the bend axis. As the angle between the bend axis and the current flow direction decreases to zero, more NWs remain intact toward the current flow. Therefore, the resulting sheet resistance of the NW network becomes smaller than when the bend axis is perpendicular to the current flow direction. This also agrees with experimental studies^34^.

**Figure 3:**
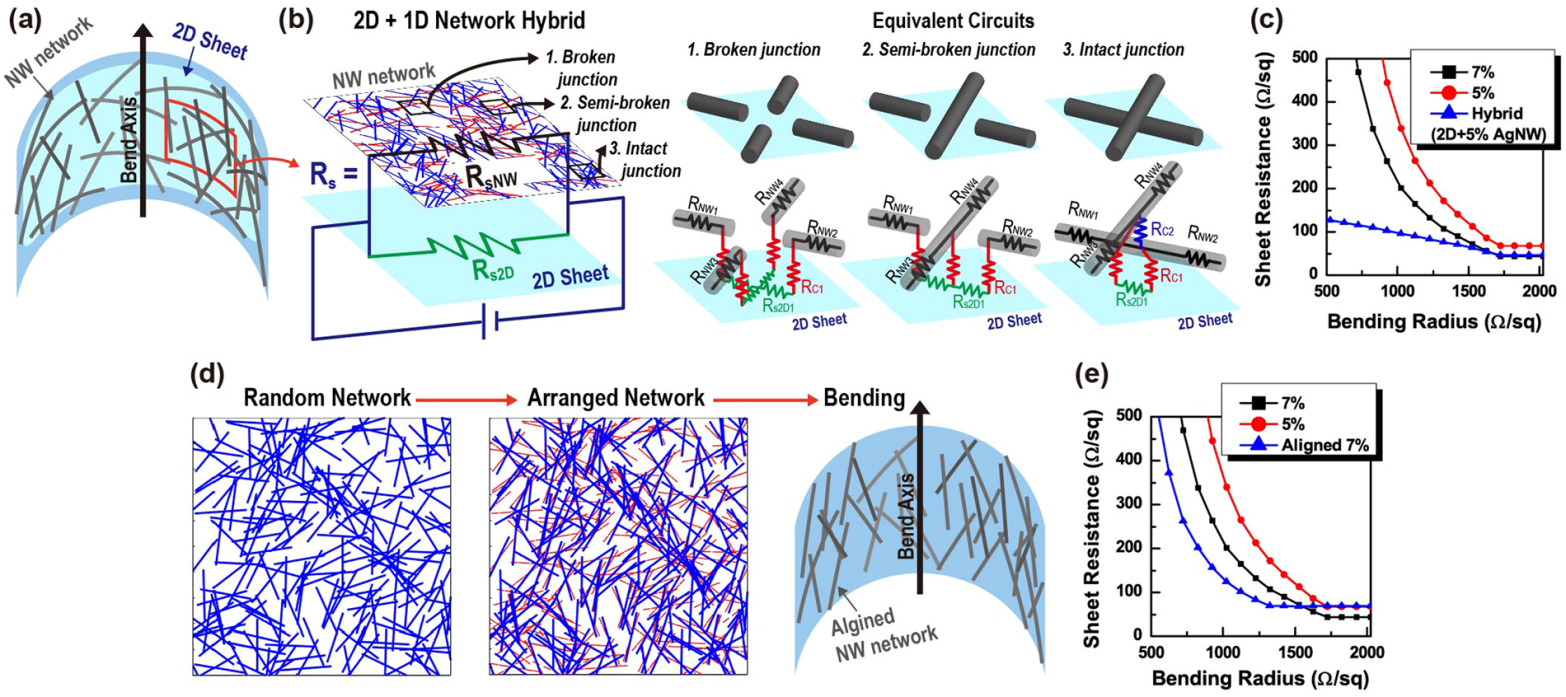
(**a**) A schematic of a hybrid of a conductive 2-D sheet (represented in sky blue color) and a NW network subjected to outward bending. (**b**) An equivalent circuit of the hybrid network in (**a**). Three types of junctions existed in the hybrid are presented along with the corresponding equivalent circuits employed in the Monte-Carlo simulation. (**c**) R_s_ of the hybrid of a 2-D sheet with R_s_ of 150 Ω/sq and a NW network with an areal coverage of 5%. For the purpose of comparison, R_s_ values of the NW network of 7% and 5% areal coverage are also presented in the same plot. (**d**) Simulation images of a random network (1st panel) and an aligned network (2nd panel) of NWs. The aligned network is obtained by rotating the NWs in the random network toward the bend axis. Red dashed lines in the 2nd panel represent the original position of the NWs (blue line) before the rotation. (**e**) R_s_ of the aligned NW network with an areal coverage of 7% as a function of bending radius. For the purpose of comparison, R_s_ values of the randomly distributed NW network of 7% and 5% areal coverage are also presented in the same plot.

A hybrid system of the NW network and conductive 2-D sheet, as described in Fig. 3(a), is exploited using a Monte-Carlo based model to confirm quantitatively the enhanced robustness of R_s_ to the bending strain of the approach^29^. According to previous studies, a NW random network coated on a flexible, transparent, and conductive 2-D sheet is capable of forming a co-percolating network such that the 2-D sheet links disconnected NWs via bypassing routes^3,29,42^. The co-percolating network is, in simulation, modeled in an equivalent resistor circuit described in Fig. 3(b). In the equivalent circuit, two major component resistors that originate from the NW network (R_sNW_) and the square 2-D sheet (R_s2D_) are connected in parallel to contribute to the total sheet resistance of the hybrid system (R_s_). R_s2D_ is the sheet resistance of the whole 2-D material plane. R_sNW_ of the NW network coated on top of the 2-D sheet, where some wires are broken due to strain (represented in red lines in the NW network in Fig. 3(b)) and others remain intact (represented in blue lines in the NW network in Fig. 3(b)), is obtained by considering equivalent circuits of three different junctions. In the NW network lying on the 2-D sheet, bending strain-induced broken junctions of NWs can remain connected by the 2-D sheet to form a co-percolating network. Therefore, in the equivalent circuit, the contact resistance between a single NW and the 2-D sheet (R_c1_, represented in a red resistance symbol) is introduced in connection with bypassing routes via the 2-D sheet. In a bypassing route, a resistor connecting contact resistors (R_s2D1_) is associated with the resistance of the 2-D sheet where the current flows. Since R_s2D1_ is very small compared to R_c1_ and is connected in series with it, this resistance can be neglected in the calculation. In the case of an intact junction, most of the current flows through the contact between two NWs since the contact resistance between them (R_c2_, represented in a blue resistance symbol) is much less than R_c1_. The contact resistance between a single NW and the 2-D sheet (R_C1_) is set to1000 Ω/sq, twice of the contact resistance between NWs (R_C2_), while R_s2D_ of the 2-D sheet is set to 150 Ω/sq, comparable to the sheet resistance of highly doped single layer graphene^43,44^. The sheet resistance change of the hybrid network with the areal coverage of 5% can be compared with the NW random network with the areal coverage of 7% since R_s0_ of both networks are similar. As presented in Fig. 3(c), the robustness to the bending deformation of the hybrid system enhances remarkably over the NW network. Additional results from the simulation with various values of R_C1_ are provided in Supplementary Information S3 and show the improved strain tolerance of the hybrid system. The simulation results show good consistency with experimental results of a hybrid network of Ag NWs and monolayer graphene, exhibiting the electro-mechanical stability superior to a bare Ag NW network under a long-period bending-fatigue test^29^.

Based on the numerical analysis, a unidirectionally aligned NW network is proposed to improve the robustness of R_s_ to the bending strain, and the enhanced performance is demonstrated using a quantitative model. As seen from blue bars in the right panel of Fig. 3(d), the NWs forming a random network can be unidirectionally assembled on the substrate^30^ or aligned toward the bend axis by the post-fabrication treatment such as unidirectional stretching^45^. The alignment of NWs along the bend axis can reduce tensile strain induced to the NW under outward bending, leading to the enhancement in the bending tolerance of the NW network. In simulation, the aligned network (the right panel of Fig. 3(a)) is obtained by modifying the angle of NWs in the random network (the left panel of Figure. 3(a), *θ*_0_ = 0 ~ π) to *θ*′ = 0.7*θ*_0_ + 0.15π. Figure 3(e) compares the sheet resistance change of the aligned NW network with 7% areal coverage to a random network with the same coverage under various configurations of the bending strain. Although the initial resistance (R_s0_, without bending strain) of the aligned NW network evaluated from the electric current flowing toward the perpendicular direction of the bend axis is larger than the resistance value of the random network, the sheet resistance change under the bending strain decreases appreciably, resulting in a smaller value of R_s_ at a smaller bending radius.

**Figure 4:**
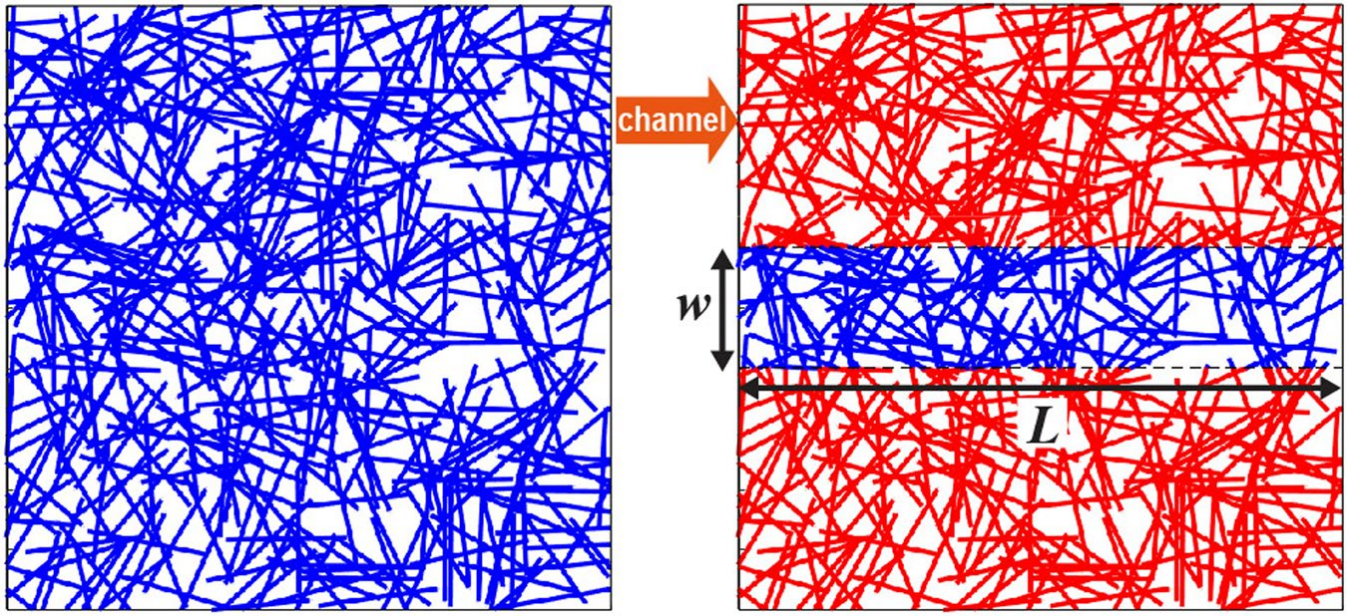
Simulation images of NW networks. A NW network randomly generated in a square domain (left panel) is deformed to a narrow channel with a width of *w* and a length of *L* (right panel). To define the channel, all NWs located outside the channel area (red NWs) are removed from the simulation.

Another aspect is the sheet resistance degradation of the NW random network patterned into a narrow channel with small feature size on the order of micrometer. Figure 4 illustrates the model considered in simulation. After constructing a NW percolating network in a square domain (the left panel in Fig. 4), a narrow channel is defined across the center of the network by eliminating all NWs out of the channel area (the right panel in Fig. 4). For channel patterning, the simulation considers currently available patterning fabrication method applied to NW networks, e.g., the photolithography followed by etching, UV exposure upon pattern mask, and laser patterning^46^. Some NWs of the resulting network can be located off the region of the channel but crossing the edge of the channel. The resistance of such a NW is prorated according to the length included in the channel. Note that R_s_ of the narrow channel is evaluated from the relationship of ${R}_{s}={R}_{Ch}\times (w/L)$, where R_*Ch*_ is the resistance of the network patterned into the channel with width *w* and length *L* estimated from the electric current flowing from the left to the right along the channel.

**Figure 5:**
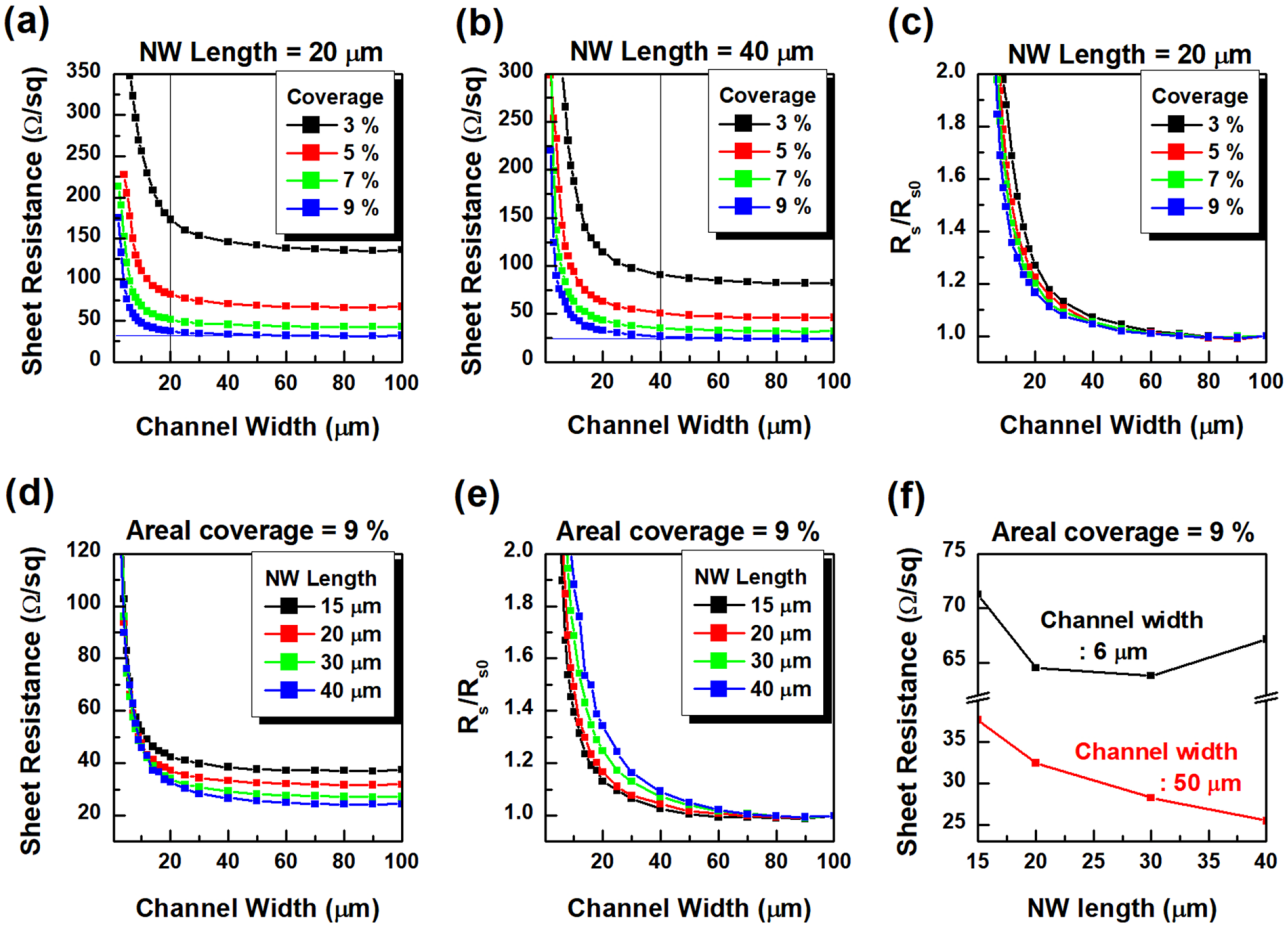
R_s_ *versus* channel width with respect to variation in areal coverages of (**a**) 20 μm length NW networks and (**b**) 40 μm length NW networks. (**c**) The relative changes of R_s_ (R_s_/R_s0_) of 20 μm length NW networks as a function of channel width. (**d**) R_s_ and (**e**) R_s_/R_s0_ the NW networks with areal coverage of 9% as a function of channel width for various NW lengths. (**f**) R_s_ of the NW networks with areal coverage of 9% for various NW lengths in the case of channel width of 50 μm and 6 μm.

Figure 5(a,b) show the sheet resistance of the percolation network comprised of 30 nm diameter NWs with lengths of 20 μm and 40 μm, respectively, with respect to the channel width for various areal coverages of the NW network. The results clearly show that R_s_ of the patterned NW network increases significantly, if the width of the channel is similar to or less than the length of the ingredient NWs of the network, and yield a good match with experimental results^47^. The relative changes in R_s_ (R_s_/R_s0_, R_s0_ refers to R_s_ of the unpatterned network) calculated from the results in Fig. 5(a) are presented in Fig. 5(c). R_s_ of the NW network with lower areal coverage is more susceptible to the channel with the reduction. With a fixed areal coverage, a NW network with longer NWs yields a larger variation in the value of R_s_ depending on the channel width. According to the results in Fig. 5(d) and (e), a longer NW network has a rapid increase for a decreasing channel width. Therefore, the longest NW is not necessarily the best choice to achieve the lowest sheet resistance of the percolating network if the NW network is fabricated into narrow patterns. In particular, R_s_ of the NW network decays as the length of the component NWs increases for the channel width of 50 μm, as shown in Fig. 5(f). If the channel width is sufficiently large, a decreasing value of R_s_ is obtained with longer NWs, showing the consistency with the tendency for R_s_ of the NW network. On the other hand, if the NW network is patterned into 6 μm wide channel, 30 μm NW network provides a smaller value of R_s_ than 40 μm NW network. For a given areal coverage of NWs, a long NW network contains a smaller number of NWs than a short NW network. If those networks are patterned into a relatively narrow width channel with respect to the length of the NW, the average length of the NWs that lie on the channel depends on the patterned channel width. A large portion of the NWs crossing the channel break down into several pieces and the remaining pieces in the channel have an average length similar to the channel width. Thus, the resulting average length of the NWs in each NW network becomes similar. As a result, a long NW network that contains a smaller number of NWs provides a larger value of R_s_ than a short NW network if the patterned channel width is relatively small with respect to the length of a component NW.

**Figure 6:**
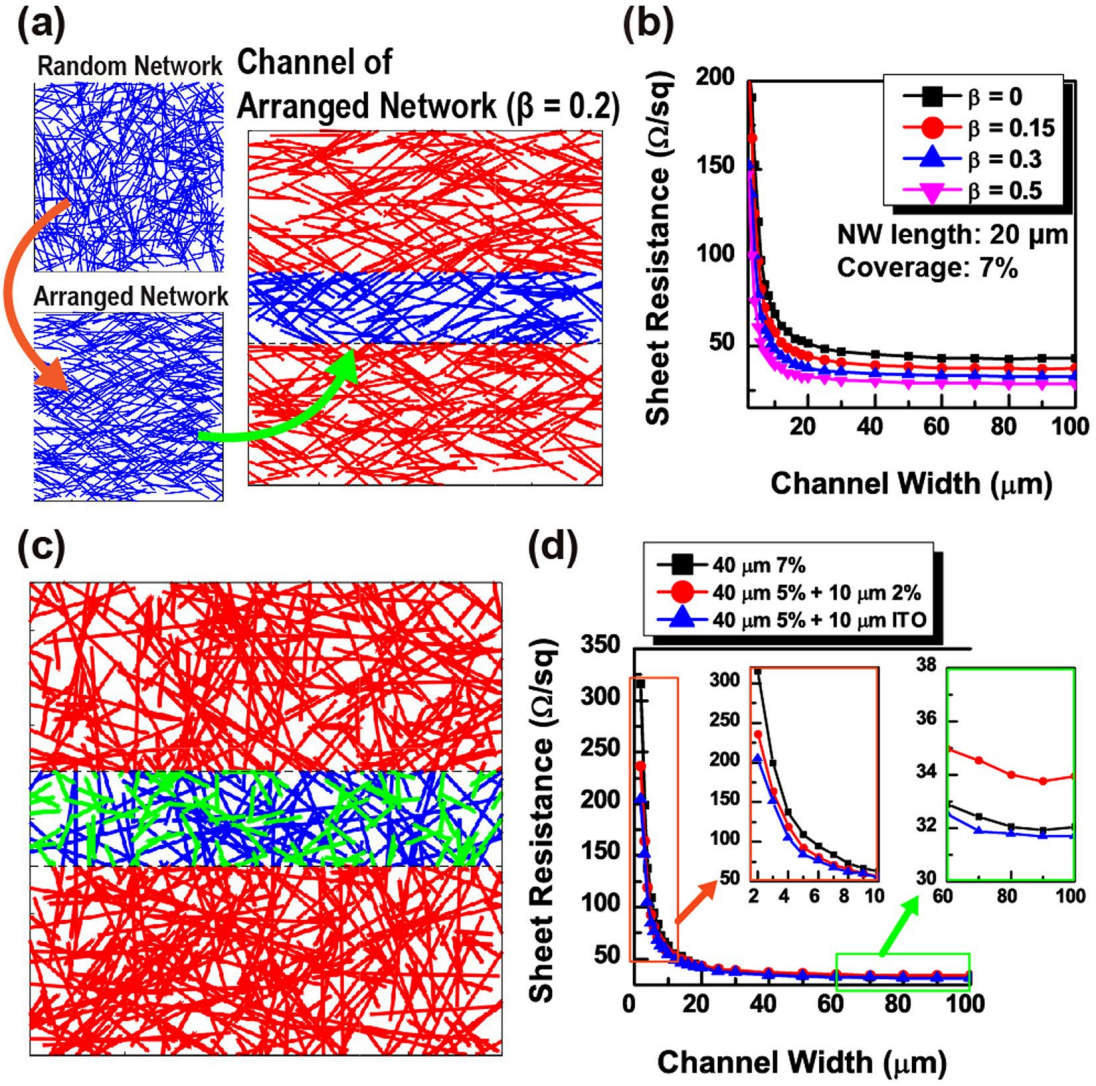
(**a**) An instance of a channel of aligned NW network. It can be obtained by unidirectionally arranging NWs in the random network, followed by defining a narrow channel. (**b**) R_s_ *versus* channel width of 20 μm NW networks with areal coverage of 7% for various degrees of alignment (β = 0, 0.15, 0.3, and 0.5). (**c**) An instance of a channel of mixed NW network comprised of NWs of two different lengths (the length of the blue NWs and green NWs are 40 μm and 10 μm, respectively) (**d**) R_s_ *versus* channel width of the mixed NW networks. For the purpose of comparison, the results of the 40 μm length NW network with areal coverage of 7% are also plotted in the same graph. Two inset graphs show some portions of the plots drawn in magnified scale for clear view of the difference between the curves.

Random NWs, which are unidirectionally assembled toward the direction parallel to a narrow channel^30^, are demonstrated in the 2-D network model in Fig. 6. The numerical results verify that a substantial increase in R_s_ by narrow channel patterning can be ameliorated. As presented in Fig. 6(a), randomly placed NWs are aligned by modifying the orientation of the NWs (*θ*_0_ = − π/2 ~ π/2) to *θ*′ = (1 − *ß*)*θ*_0_ and are followed by channel patterning. The degree of the alignment angle is determined by parameter *ß*, and three different cases (*ß* = 0.15, 0.3, and 0,5) of 20-µm NW random network with the areal coverage of 7% are considered. As shown in Fig. 6(b), the more NWs are aligned, the less sheet resistance variation with respect to the channel width are obtained. In addition, R_s0_ also decreases as the NWs are aligned further along the channel. However, this approach offers the advantage only for the narrow channel patterned to orient in a direction along the NW alignment. This anisotropic distribution of the NW orientation renders the sheet resistance of a channel patterned perpendicular to the NW alignment larger than a channel patterned onto a random NW network.

A new strategy for removing the directional anisotropy of the aligned network is to pattern a narrow channel onto a random network composed of two types of NWs with lengths 40 µm and 10 µm, as shown in Fig. 6(c). According to the results in Fig. 6(d), a random network comprised of 40 µm NWs with 5% areal coverage and 10 µm NWs with 2% areal coverage presents a smaller value of R_s_ compared to a random network of 40 µm NWs with 7% areal coverage if the width of the narrow channel is less than 20 µm. Although R_s0_ (R_s_ of unpatterned network) of the mixed type NW network is 2 Ω/sq larger than the single type NW network with the same areal coverage, R_s_ of the mixed type NW network becomes more than 16 Ω/sq, which is smaller than the single type NW network if the channel width is less than 5 µm.

For further improvement of the electrical performance of the mixed type NW network without sacrificing optical characteristics, short NWs can be replaced with 10 µm ITO NWs which have much lower optical absorption and scattering cross-section compared to metallic NWs in the visible wavelength range. However, an ITO NW has much larger resistivity. More number of ITO NWs need to be added into the network to improve electrical properties while maintaining optical performance. The material properties of the ITO NW are obtained from an experimental study^48^, where a 10 µm ITO NW grown by the standard thermal evaporation method with the diameter of 200 nm and the resistivity of 1.37 × 10^−6^ Ω·m is reported. The number of ITO NWs in the mixed type NW network increases by 1.5 times the number of 10 μm NWs used in the previous calculation. As shown in Fig. 6(d), the mixed type NW network with ITO NWs has a value of R_s0_ comparable to the single type (40 µm) NW network with 7% areal coverage. However, the mixed type network with ITO NWs presents a much smaller value of R_s_ than the single type NW network as channel width decreases. If 5 µm width channel is patterned onto the network, R_s_ of the single type NW network increases from 32 Ω/sq to 109 Ω/sq, while R_s_ of the ITO mixed type NW network increases from 32 Ω/sq to 84 Ω/sq, which corresponds to 30% less increase compared to the single type NW network.

## Conclusion

In summary, we have developed quantitative models of the electrical conductivity of the 1-D NW random network under the external deformation, and exploited strategies designed to enhance the robustness of R_s_ to bending and patterning. The tolerance of R_s_ against network bending can be improved as increasing the NW areal coverage and the length of component NWs. The performance can be further enhanced by employing a hybrid network of NWs and a conductive 2-D sheet and by arranging NWs along the direction in parallel to the bend axis. Also, the sheet resistance robustness of the NW network for a patterned narrow channel enhances as the areal coverage of NWs increases and the comprising NW length decreases. The sheet resistance robustness can be further improved by arranging the NWs in the network unidirectionally toward the direction parallel to the narrow channel and by constructing a network with a mixture of NWs with different lengths. This work can provide design guidelines for the use of the 1-D NW percolation networks in the fabrication of flexible TCEs.

## Electronic supplementary material

Supplementary Information

## Data Availability

Some of the datasets generated or analyzed during this study are included in Supplementary Information. The datasets that do not appear in Supplementary Information are available from the corresponding author on reasonable request.
