# Supplementary material for: Computational characterization and control of electrical conductivity of nanowire composite network under mechanical deformation
Source: Sci Rep. 2018 Nov 9;8:16617. doi: 10.1038/s41598-018-34992-6 (PMC6226518; doi:10.1038/s41598-018-34992-6)
Supplement: Supplementary file 1 — Supplementary Information [file 41598_2018_34992_MOESM1_ESM.docx]

**Computational characterization and control of electrical conductivity of nanowire composite network under mechanical deformation**

**Jinyoung Hwang^1^, Hiesang Sohn^2^, and Sang Hyun Lee^3,*^**

^1^ School of Electronics and Information Engineering, Korea Aerospace University, Goyang-si, 10540, Korea
^2^ Department of Chemical Engineering, Kwangwoon University, Seoul, 01897, Korea
^3^ School of Electrical Engineering, Korea University, Seoul, 02841, Korea
*sanghyunlee@korea.ac.kr

S1. Monte-Carlo Simulation


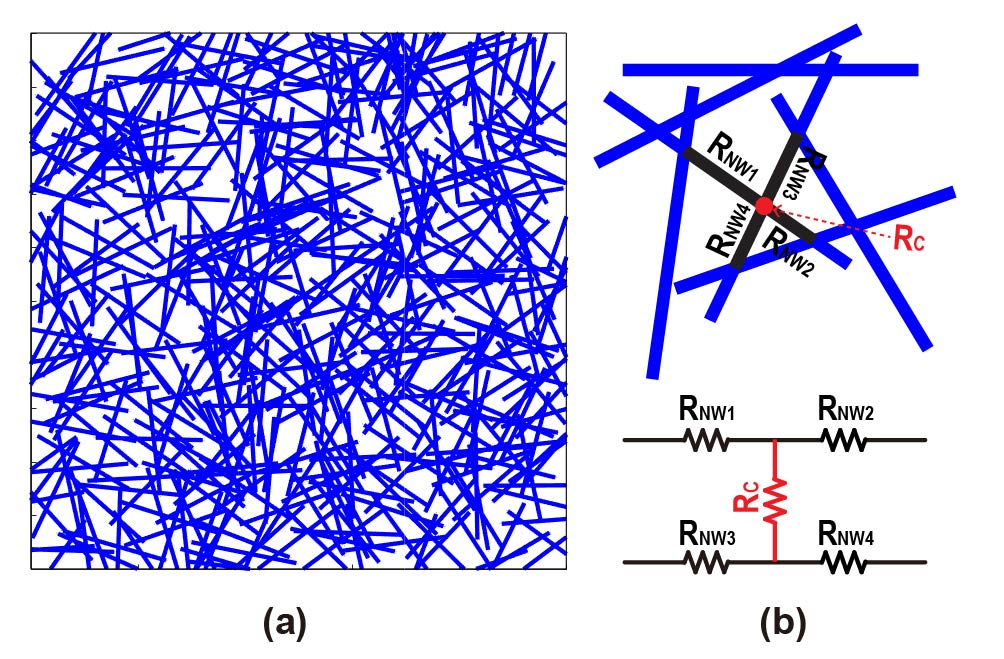


**Figure S1.** (a) An instance of NW network generated in the Monte-Carlo simulation (b) An example of a junction between NWs in the network with the corresponding equivalent circuits employed in the simulation.

A Monte-Carlo (MC) simulation is performed to predict the sheet resistance (R_s_) of a 1-D nanowire (NW) percolation network. In this simulation, a single random instance of the NW network shown in Figure S1 (a) is defined in a square domain by randomly choosing a two-dimensional center coordinate and angle of rectangular rods representing NWs. The width and length of the rod corresponds to the diameter and length of the NWs modeled as a cylindrical wire. Upon the completion of random placement of the rods, the connection configuration of the network is examined by testing the connectivity between rods, which was determined by calculating the distance between two rods. If the shortest distance between two rods is less than the width of the rods, the two rods are considered to be in contact with each other. The connectivity information is utilized to clustering analysis for exploring percolating clusters that traverse across the simulation domain. A percolating cluster that gradually expands from left end to right end of the simulation domain is considered as a conducting path. After classifying all rods into separating groups according to the percolating clusters that they belong to, wires that do not belong to any conducting path are excluded from the resistance calculation of the network. The overall resistance (R_s_) of the network is obtained based on Kirchhoff’s current law (KCL) by considering the voltage drop at every junction in the conducting paths. As the equivalent circuit in Figure S1 (b) presented, which representing the junction between NWs presented, both resistances arising from inherent NW resistance (R_NW_) and contact resistance between two NWs (R_C_) are considered. A system of linear equation can be constructed from the KCL, and the total current flow flowing across the simulation domain can be evaluated from the solution of the equation. By dividing an applied voltage across the simulation domain (set as 1 V) to the obtained total current, the R_s_ of the network can be calculated.

S2. Bending strain induced on an individual NW


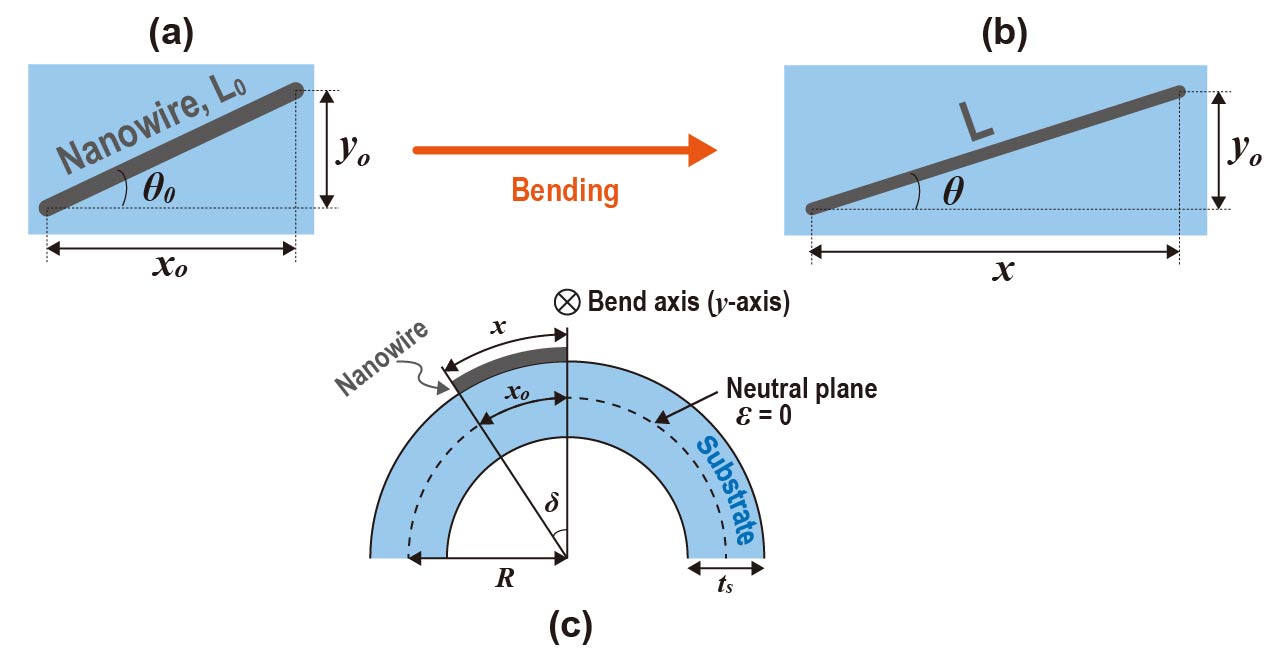


**Figure S2.** (a) A schematic description of the modification of a single NW coated on the film. (b) Under outward bending, the NW is elongated in a bending direction (perpendicular to a bend axis, *x*-axis) while the length in *y* radial direction (parallel to a bend axis) remains unchanged. (c) A cross-sectional view of the NW coated on an outwardly bend flexible substrate.

As shown in **Figure S2** (a), the lengths in *x* and *y* axes direction of a NW with the length *L*_0_ are given by

$$x_{0}=L_{0}\cos\theta_{0}$$

$$y_{0}=L_{0}\cos\theta_{0}$$

Upon outward bending along the bend axis (in parallel to *y* direction), the NW is elongated in *x*-direction while the length in *y* direction is unchanged as shown in **Figure S2** (b). The resulting lengths are given by

$$x=L\sin\theta$$

$$y=y_{0}$$

where *L* is the elongated length of the NW.

Since the neutral plane of the substrate is positioned at the half of the film thickness as presented in **Figure S2** (c),

$$x_{0}=R\delta$$

$$x=\left( R+\frac{t_{s}}{2} \right)\delta$$

where *t*_s_ and *R* represents the film thickness and the radius of curvature of the bending, respectively. Therefore, *x* can be expressed by the angle *θ_0_* and the radius of curvature *R* as follows:

$$x=\left( \frac{2R+t_{s}}{2R} \right)x_{0}=\left( \frac{2R+t_{s}}{2R} \right)L_{0}\cos\theta_{0}$$

and the elongated NW length *L* is

$$L(\theta_{0})=\sqrt{x^{2}+y_{0}^{2}}=L_{0}\sqrt{\left( \frac{2R+t_{s}}{2R} \right)^{2}\cos^{2} \theta_{0}+\sin^{2} \theta_{0}}$$

With the above expression, the bending strain induced on the NW can be written by

$$\varepsilon\left( \theta_{0} \right)=\frac{L(\theta_{0})-L_{0}}{L_{0}}=\sqrt{\left( \frac{2R+t_{s}}{2R} \right)^{2}\cos^{2} \theta_{0}+\sin^{2} \theta_{0}}-1$$

S3. R_s_ of the hybrid of 2-D sheet and a NW network


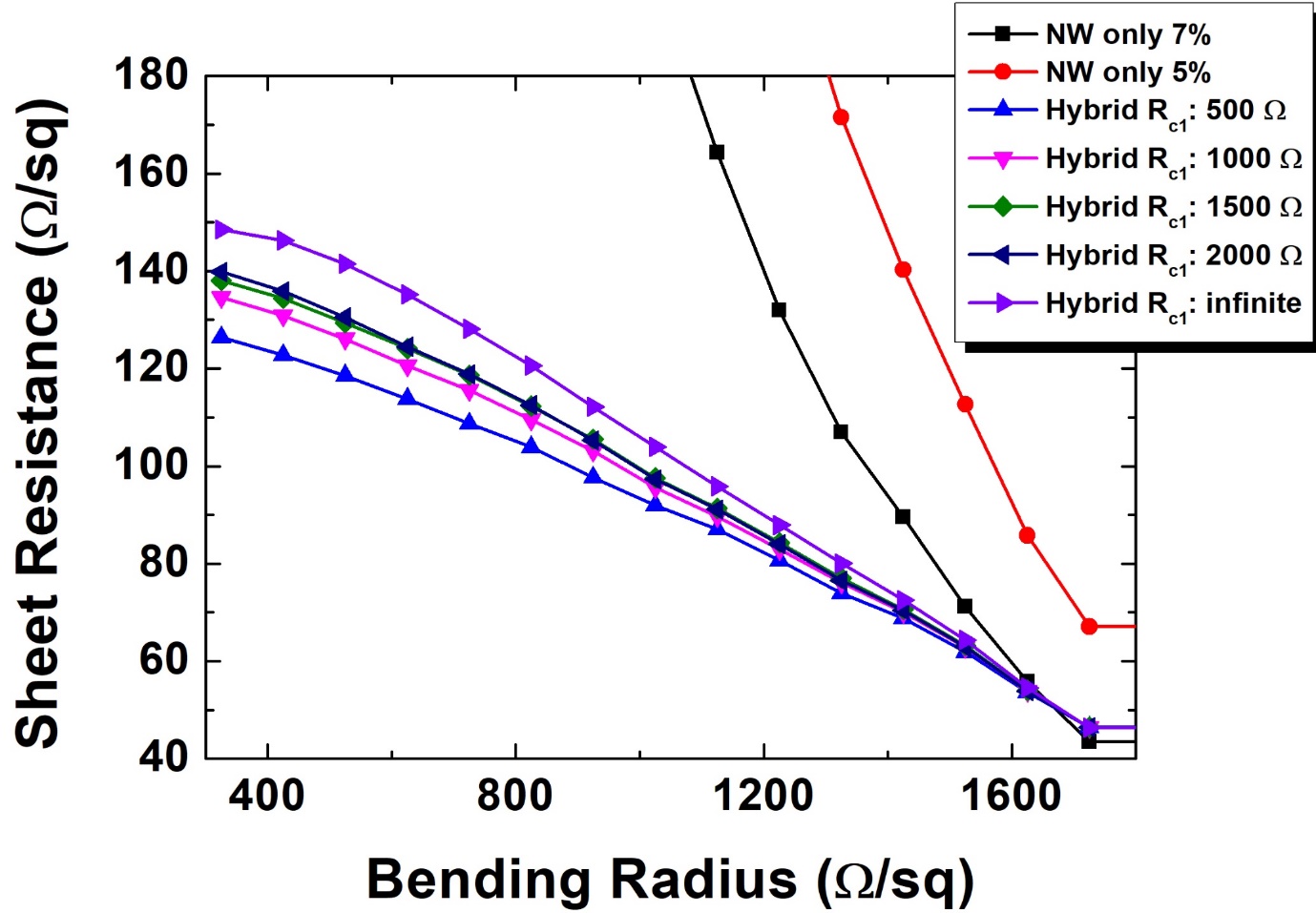


**Figure S3**. R_s_ of the hybrid of a 2-D sheet with R_s_ of 150 Ω/sq and a NW network of 5 % areal coverage with respect to variation in the values of contact resistance between a single NW and the 2-D sheet (R_C1_). For the purpose of comparison, R_s_ values of the NW network of 7 % and 5 % areal coverage are also presented in the same plot.

S4. R_s_ of the nanowire network for various configurations of the bending axis angle.


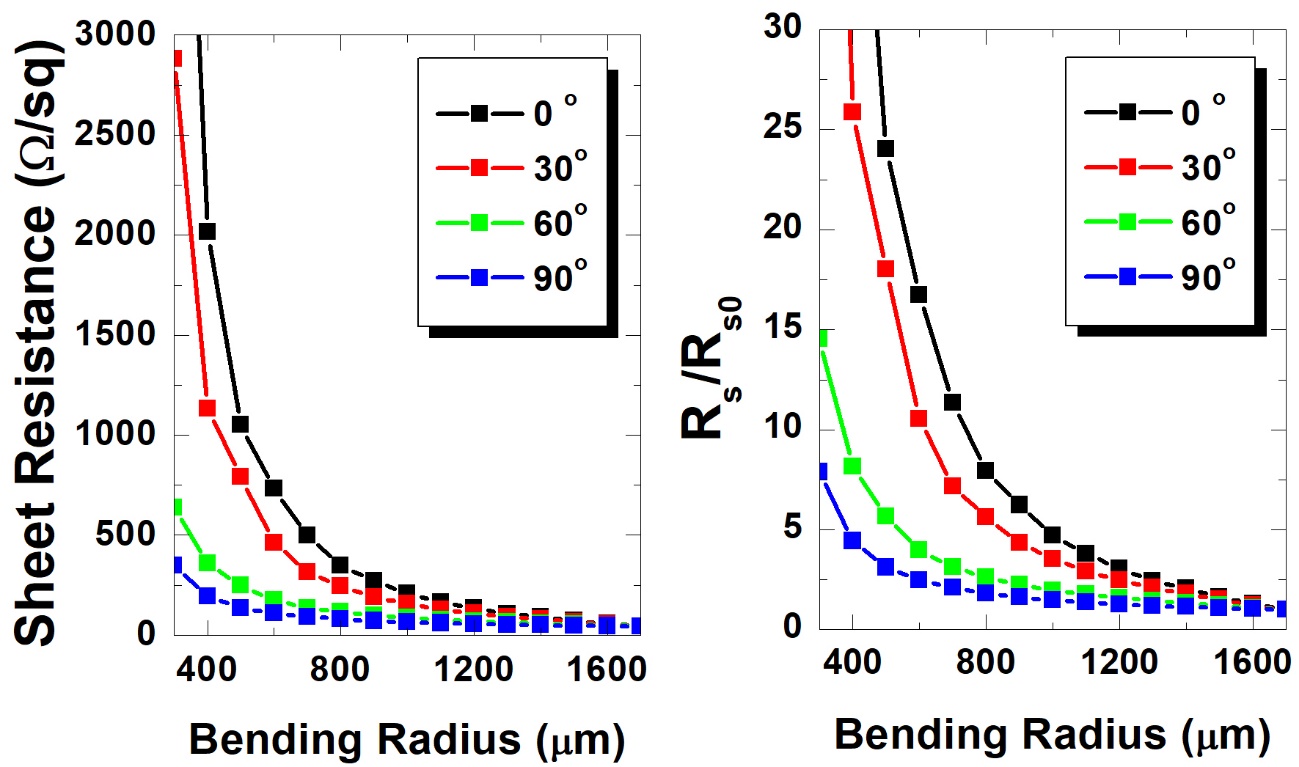


**Figure S4**. R_s_ of the hybrid of the network with 20 μm NWs and 7% areal coverage as a function of bending radius corresponding to the angle of bending axis. When the bending axis is aligned to *y*-axis and *x*-axis (the axis is indicated in figure 1 (a)), the angle of bending axis is 0° and 90°, respectively. The sheet resistance is calculated based on the electrical current flowing in *x*-axis.
